# Supplementary material for: Ladder Use in Older People: Type, Frequency, Tasks and Predictors of Risk Behaviours
Source: Int J Environ Res Public Health. 2021 Sep 17;18(18):9799. doi: 10.3390/ijerph18189799 (PMC8471785; doi:10.3390/ijerph18189799)
Supplement: Supplementary file 1 [file ijerph-18-09799-s001.zip › ijerph-1331379-SI.pdf]

Supplementary Table S1. Participants' reported step, straight, and fixed ladder use (frequency, tasks, locations, and climbing heights) for men, women, and the total sample (number of responses and percentages).

	Step Ladder			Straight Ladder			Fixed Ladder		
	**Men**	**Women**	**Total**	**Men**	**Women**	**Total**	**Men**	**Women**	**Total**
**Ladder use frequency**	***n* = 51**	***n* = 51**	***n* = 102**	***n* = 51**	***n* = 51**	***n* = 102**	***n* = 51**	***n* = 51**	***n* = 102**
Never	2 (3.9)	2 (3.9)	4 (3.9)	13 (25.5)	31 (60.8)	44 (43.1)	29 (56.9)	38 (74.5)	67 (65.7)
Once per year	1 (2.0)	3 (5.9)	4 (3.9)	7 (13.7)	6 (11.8)	13 (12.7)	10 (19.6)	4 (7.8)	14 (13.7)
Few times per year	16 (31.4)	16 (31.4)	32 (31.4)	17 (33.3)	13 (25.5)	30 (29.4)	10 (19.6)	8 (15.7)	18 (17.6)
Monthly	13 (25.5)	16 (31.4)	29 (28.4)	10 (19.6)	0 (0)	10 (9.8)	1 (2.0)	1 (2.0)	2 (2.0)
Weekly	11 (21.6)	7 (13.7)	18 (17.6)	3 (5.9)	0 (0)	3 (2.9)	1 (2.0)	0 (0)	1 (1.0)
Few times per week	8 (15.7)	7 (13.7)	15 (14.7)	1 (2.0)	1 (2.0)	2 (2.0)	0 (0)	0 (0)	0 (0)
**Ladder use tasks**	***n* = 49**	***n* = 49**	***n* = 98**	***n* = 38**	***n* = 20**	***n* = 58**	***n* = 22**	***n* = 13**	***n* = 35**
Changing a light bulb	43 (87.8)	26 (53.1)	69 (70.4)	1 (2.6)	2 (10.0)	3 (5.2)	0 (0)	0 (0)	0 (0)
Cleaning the gutters	10 (20.4)	2 (4.1)	12 (12.2)	28 (73.7)	8 (40.0)	36 (62.1)	0 (0)	0 (0)	0 (0)
Washing the windows	18 (36.7)	16 (32.7)	34 (34.7)	12 (31.6)	7 (35.0)	19 (32.8)	0 (0)	0 (0)	0 (0)
Cutting branches/Picking fruit	19 (38.8)	11 (22.4)	30 (30.6)	15 (39.5)	7 (35.0)	22 (37.9)	0 (0)	0 (0)	0 (0)
Getting objects from attic	9 (18.4)	6 (12.2)	15 (15.3)	1 (2.6)	2 (10.0)	3 (5.2)	6 (27.3)	3 (23.1)	9 (25.7)
Decorating	25 (51.0)	17 (34.7)	42 (42.9)	9 (23.7)	5 (25.0)	14 (24.1)	2 (9.1)	2 (15.4)	4 (11.4)
Getting objects from high shelves *	6 (12.2)	18 (36.7)	24 (24.5)	0 (0)	0 (0)	0 (0)	0 (0)	0 (0)	0 (0)
Entering/Exiting swimming pool *	0 (0)	0 (0)	0 (0)	0 (0)	0 (0)	0 (0)	7 (31.8)	6 (46.2)	13 (37.1)
Accessing a boat *	0 (0)	0 (0)	0 (0)	0 (0)	0 (0)	0 (0)	5 (22.7)	4 (30.8)	9 (25.7)
Other	7 (14.3)	2 (4.1)	9 (9.2)	9 (23.7)	1 (5.0)	10 (17.2)	7 (31.8)	0 (0)	7 (20.0)
**Ladder use locations**	***n* = 49**	***n* = 49**	***n* = 98**	***n* = 38**	***n* = 20**	***n* = 58**	***n* = 22**	***n* = 13**	***n* = 35**
Inside	84 (85.7)	43 (87.8)	41 (83.7)	12 (20.7)	7 (18.4)	5 (25.0)	5 (14.3)	2 (9.1)	3 (23.1)
Outside	54 (55.1)	34 (69.4)	20 (40.8)	52 (89.7)	34 (89.5)	18 (90.0)	14 (40.0)	7 (31.8)	7 (53.8)
At home	75 (76.5)	38 (77.6)	37 (75.5)	44 (75.9)	30 (78.9)	14 (70.0)	8 (22.9)	5 (22.7)	3 (23.1)
Other than home	8 (8.2)	7 (14.3)	1 (2.0)	8 (13.8)	6 (15.8)	2 (10.0)	21 (60.0)	14 (63.6)	7 (53.8)
**Highest ladder climbing height**	***n* = 49**	***n* = 49**	***n* = 98**	***n* = 38**	***n* = 20**	***n* = 58**	***n* = 22**	***n* = 13**	***n* = 35**

1st step	0 (0)	0 (0)	0 (0)	1 (1.7)	0 (0)	1 (5.0)	0 (0)	0 (0)	0 (0)
Below halfway	9 (9.2)	3 (6.1)	6 (12.2)	3 (5.2)	1 (2.6)	2 (10.0)	1 (2.9)	0 (0)	1 (7.7)
Halfway	21 (21.4)	10 (20.4)	11 (22.4)	10 (17.2)	5 (13.2)	5 (25.0)	3 (8.6)	1 (4.5)	2 (15.4)
Above halfway	68 (69.4)	36 (73.5)	32 (65.3)	44 (75.9)	32 (84.2)	12 (60.0)	31 (88.6)	21 (95.5)	10 (76.9)

* These activities were not specifically queried in the survey but were included in free text for "other uses".
